# Testicular Embryonal Carcinoma With Retroperitoneal Metastasis and Normal AFP and HCG: A Case Report

**DOI:** 10.1155/crom/9821202

**Published:** 2026-04-07

**Authors:** Panpan Teng, Mingxi Yang, Shang Song

**Affiliations:** ^1^ Department of Urology Surgery, Tongren People’s Hospital, Guizhou, China

**Keywords:** case report, embryonal carcinoma, testicular neoplasms, tumor markers

## Abstract

Testicular germ cell tumors are common malignant tumors in young men, among which embryonal carcinoma, as an important subtype of non‐seminomatous germ cell tumors, is usually accompanied by elevated serum tumor markers. This case report describes a 24‐year‐old male patient with testicular embryonal carcinoma and retroperitoneal lymph node metastasis, yet with persistently normal serum markers, posing significant diagnostic challenges. Diagnosis was ultimately confirmed through imaging, histopathology, and immunohistochemical analysis. The patient underwent radical orchiectomy followed by standard bleomycin, etoposide, and cisplatin (BEP) chemotherapy and showed no recurrence at 12‐month follow‐up. This case highlights the necessity of a multidisciplinary diagnostic approach, integrating clinical evaluation, imaging, and immunohistochemistry, to ensure accurate diagnosis, particularly in marker‐negative presentations. It also confirms the efficacy of established treatment protocols and emphasizes the importance of heightened clinical vigilance and long‐term follow‐up for such atypical cases.

## 1. Introduction

Testicular embryonal carcinoma is a rare but highly aggressive subtype of non‐seminomatous germ cell tumors (NSGCTs), predominantly affecting young adult males, with a peak incidence between 20 and 30 years of age and accounting for approximately 2%–3% of all testicular tumors [1, 2]. Embryonal carcinoma is characterized by a rapid growth rate and a significant propensity for early metastatic spread, most commonly to the retroperitoneal lymph nodes, which is a hallmark of advanced disease at initial presentation [1, 3]. The typical clinical manifestation is a painless, progressively enlarging testicular mass; however, because of the lack of pain or discomfort, early detection is often delayed, potentially resulting in diagnosis at a more advanced stage [1, 4].

Diagnosis of testicular germ cell tumors generally relies on a combination of clinical findings, imaging, serum tumor markers, and histopathology. The measurement of alpha‐fetoprotein (AFP) and beta‐human chorionic gonadotropin (*β*‐HCG) is central to the diagnostic algorithm, as these markers are elevated in the majority of NSGCTs and are valuable for both staging and monitoring response to therapy [1]. However, a notable subset of embryonal carcinomas presents with normal AFP and *β*‐HCG levels, which complicates the clinical diagnostic process and increases the risk of initial misclassification as seminoma or other less aggressive neoplasms [1, 2].

This case is of particular interest because it features a young male with a classic presentation of a painless, progressively enlarging testicular mass, but with persistently normal serum AFP and *β*‐HCG, in the context of clear radiological and pathological evidence of metastatic retroperitoneal lymphadenopathy. Such cases are diagnostically challenging and may be at risk for delayed or inappropriate management if clinicians rely exclusively on serum markers for initial stratification [1, 2]. The clinical value and novelty of this case lie in its demonstration that embryonal carcinoma with normal serum tumor markers can present with advanced metastatic disease, underscoring the need for a comprehensive, multidisciplinary diagnostic approach that does not exclude NSGCTs in marker‐negative patients.

## 2. Case Presentation

A 24‐year‐old male was admitted on April 7, 2022, due to a right testicular mass that had been present for 6 months. The patient reported that he had noticed a pea‐sized mass in the right testis 6 months earlier, with no pain symptoms, and did not pay attention to it. Subsequently, the mass gradually enlarged, leading him to seek medical attention at our hospital. Physical examination showed that the right testis was larger than the left, with a hard mass approximately 2 × 2 cm in size, and no tenderness was noted.

**Figure 2:**
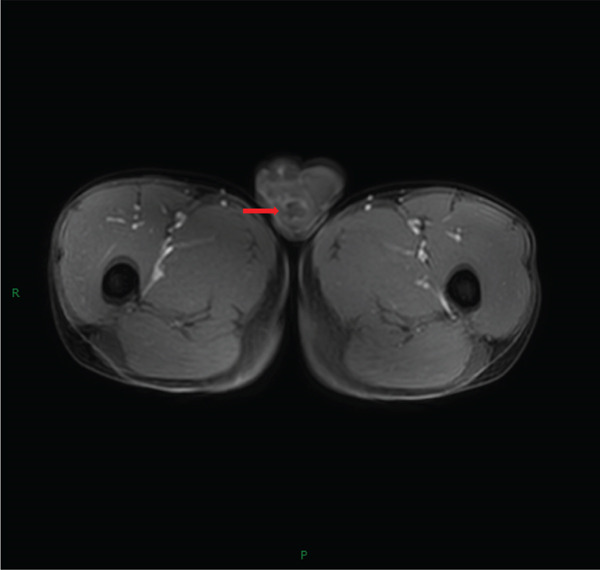
MRI findings of a right testicular mass. Post‐contrast fat‐suppressed T1‐weighted image reveals heterogeneous enhancement (arrow).

**Figure 3:**
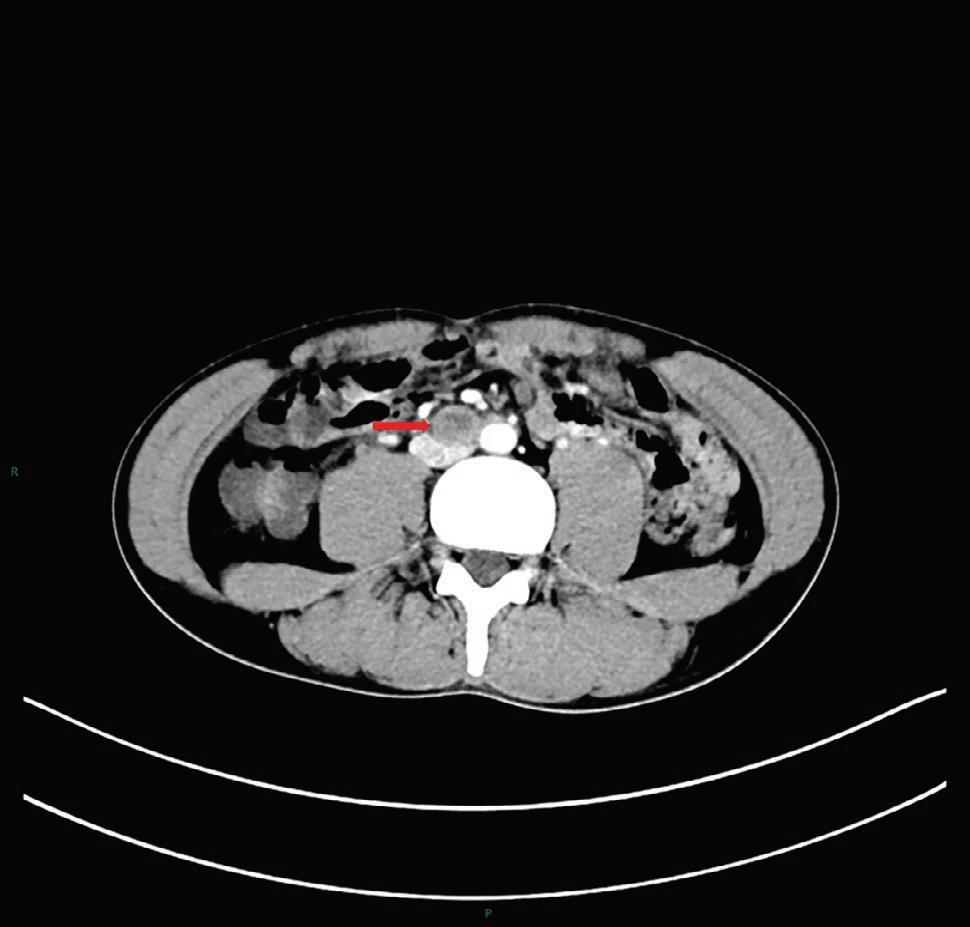
Abdominal CT image showing multiple, partially enlarged retroperitoneal lymph nodes with heterogeneous ring enhancement post‐contrast administration (arrow).

Laboratory tests showed that AFP was 4.2 ng/mL, and *β*‐HCG was < 0.1 mIU/mL, both within the normal range. MRI examination showed irregular enlargement of the right testis with nodular mass shadows, T1WI showing iso‐ to slightly high signal (Figure 1a), and T2WI showing iso‐ to slightly low signal (Figure 1b), measuring approximately 1.7 × 1.6 × 1.5 cm, with enhanced scan showing mild uneven enhancement (Figure 2). Abdominal CT showed multiple retroperitoneal lymph node shadows, with the largest diameter approximately 19 mm, showing uneven ring enhancement (Figure 3). The preliminary diagnosis was right testicular seminoma.

Figure 1MRI findings of a right testicular mass. (a) Axial T1‐weighted image shows an irregularly bordered mass with iso‐ to slightly hyperintense signal (arrow). (b) Axial T2‐weighted image demonstrates iso‐ to slightly hypointense signal relative to normal parenchyma (arrow).

**Figure:**
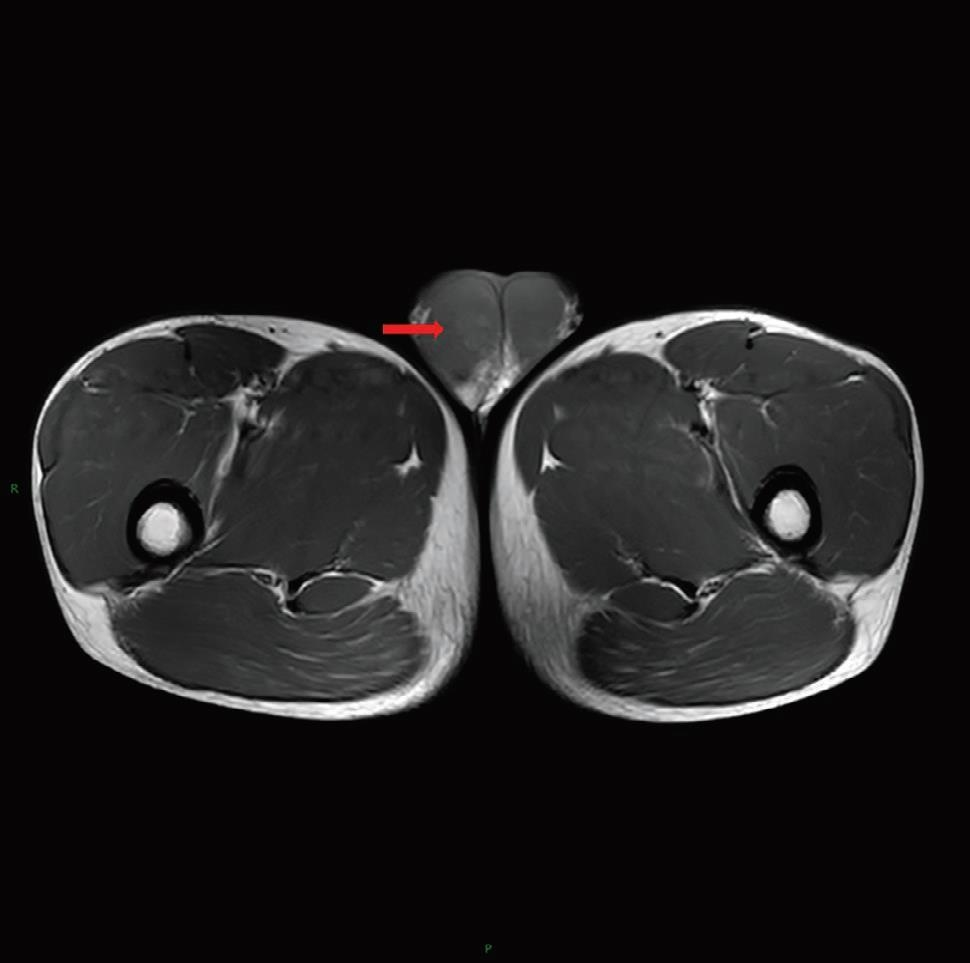
(a)

**Figure:**
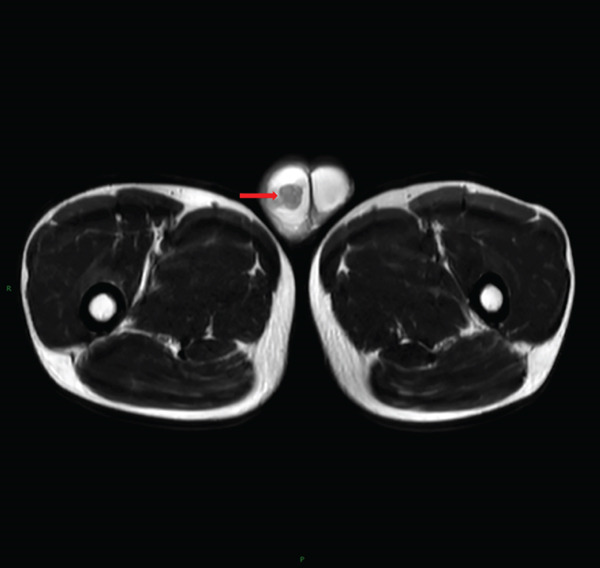
(b)

**Figure 4:**
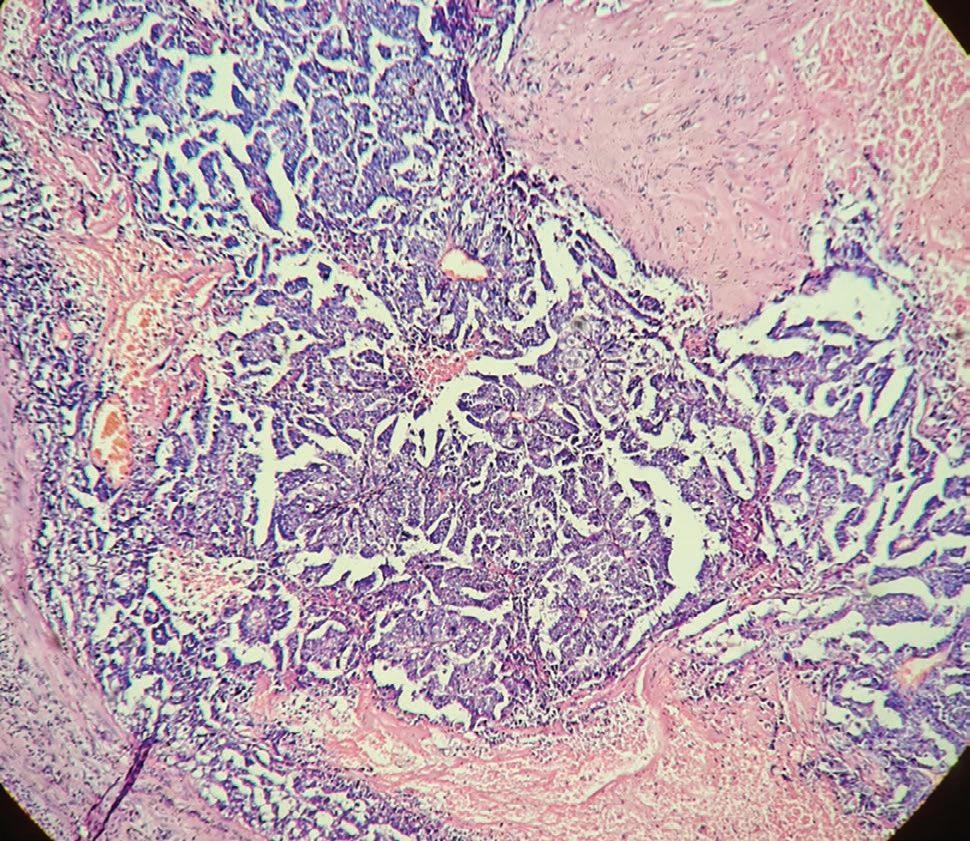
Pathological features of embryonal carcinoma of the right testis. (HE, × 200). The tumor cells exhibit a nested and sheet‐like growth pattern with clear cytoplasm, focally forming glandular and reticular structures.

The patient underwent right radical orchiectomy, during which the tunica albuginea was intact, the tumor boundary was clear, and necrotic foci were visible internally. Postoperative pathological examination showed that tumor cells exhibited translucent nest‐like growth, with local glandular and reticular structures (Figure 4).

Immunohistochemical results showed the following: CK‐pan (+), CD30 (+++), Ki‐67 (70%), PLAP (+), AFP (focal +), consistent with the diagnosis of embryonal carcinoma (Figure 5). Postoperative PET‐CT examination showed multiple enlarged retroperitoneal lymph nodes, with the largest approximately 2.4 × 2.7 cm, with increased metabolism, suggesting metastasis.

Figure 5Embryonal carcinoma of the right testis. (a) Strong membranous CD30 positivity; (b) Focal cytoplasmic AFP positivity. (IHC, × 200).

**Figure:**
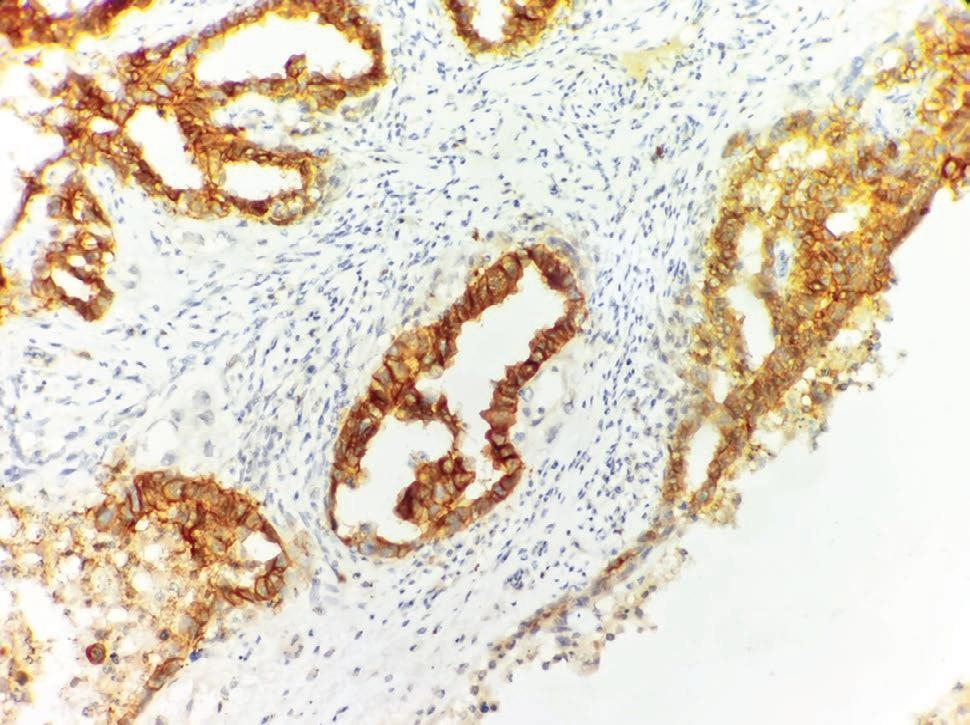
(a)

**Figure:**
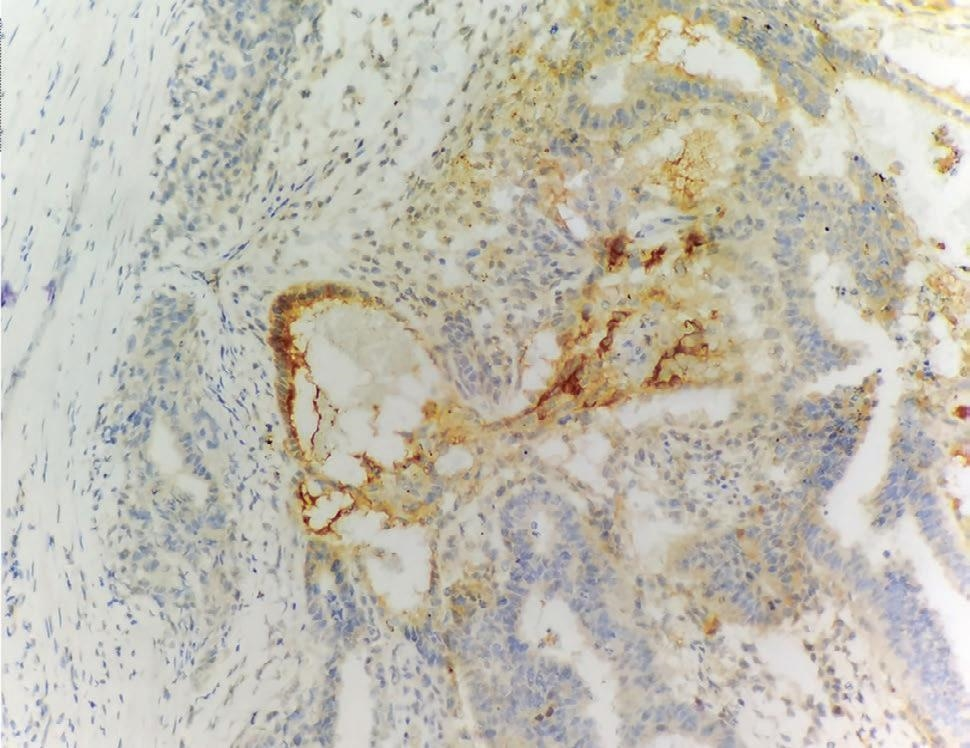
(b)

Based on the AJCC TNM classification (8th edition), the patient was staged as pT1N2M0S0 (Stage IIB) following radical orchiectomy and imaging. According to the IGCCCG risk classification for non‐seminoma, with normal serum markers (S0) and non‐pulmonary visceral metastases absent, the patient was classified as having a good prognosis.

Following surgery, the patient received four cycles of adjuvant chemotherapy using the BEP regimen, consisting of cisplatin, etoposide, and bleomycin. The patient tolerated the treatment well without severe adverse effects.

After completion of chemotherapy, repeat abdominal CT demonstrated significant reduction in the size of the retroperitoneal lymphadenopathy (Figure 6a). During a 12‐month follow‐up period, regular abdominal CT scans showed no further enlargement of the retroperitoneal lymph nodes (Figure 6b). Serum tumor markers (AFP and *β*‐HCG) remained within normal limits at all subsequent assessments (Table 1). The patient remained in good general condition with no evidence of tumor recurrence.

Figure 6Abdominal computed tomography (CT) evaluation of retroperitoneal lymph nodes after chemotherapy and during follow‐up. (a) Axial contrast‐enhanced CT after chemotherapy shows multiple retroperitoneal lymph nodes with residual enlargement but significant reduction in size compared to pre‐chemotherapy findings (arrow). (b) Follow‐up axial CT at 12 months after chemotherapy completion demonstrates no further enlargement of the retroperitoneal lymph nodes, indicating stable disease (arrow).

**Figure:**
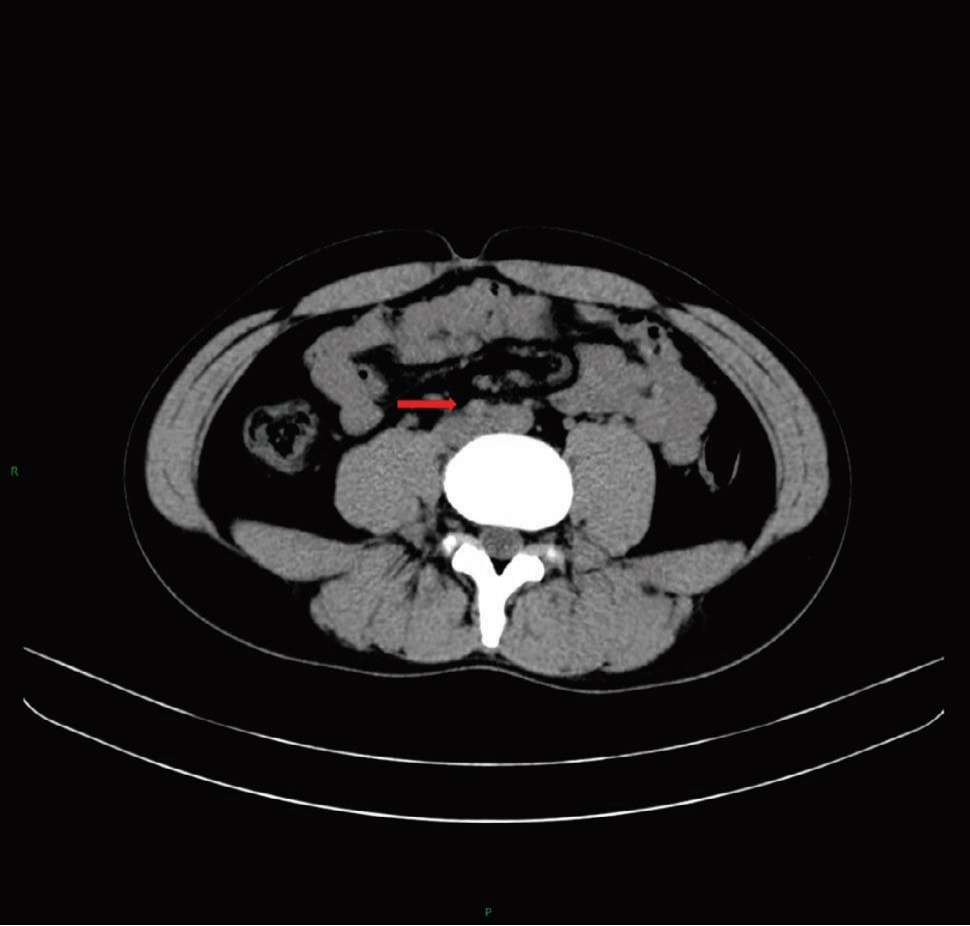
(a)

**Figure:**
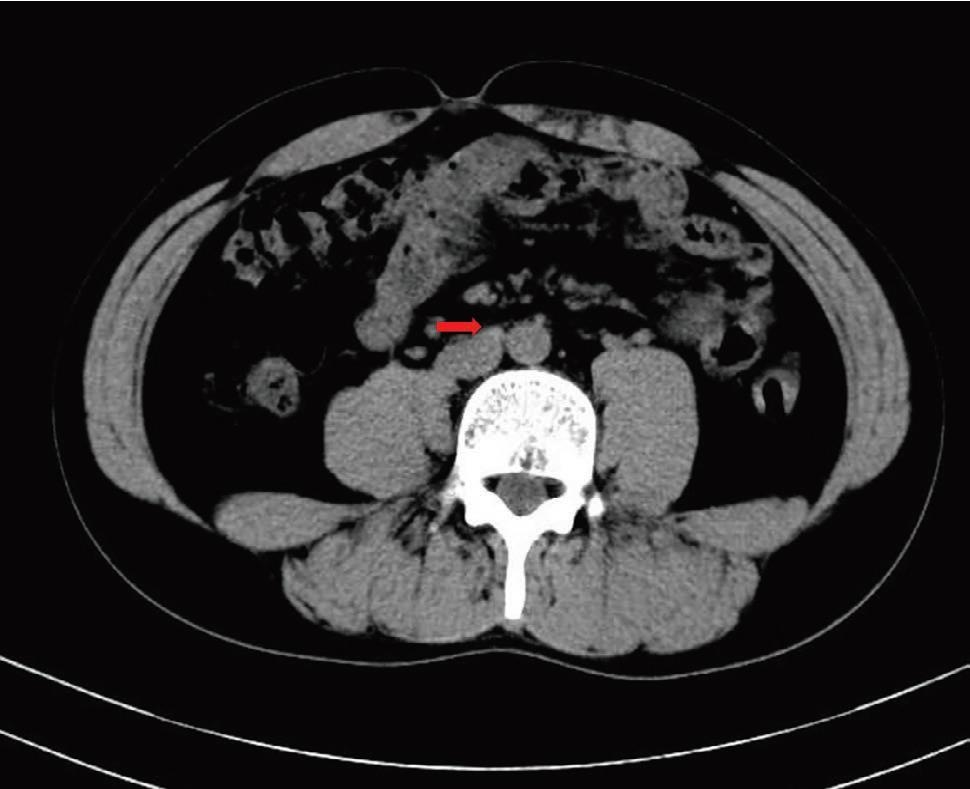
(b)

**Table 1:** Serial serum tumor marker levels during diagnosis, treatment, and follow‐up.

Time point	AFP (ng/mL)	*β*‐HCG (mIU/mL)	Clinical context
At initial diagnosis	4.2	<0.1	Pre‐orchiectomy
Post‐orchiectomy	4.5	< 0.1	Pre‐chemotherapy
After BEP cycle 1	4.3	< 0.1	During chemotherapy
After BEP cycle 2	4.6	< 0.1	During chemotherapy
After BEP cycle 3	4.4	< 0.1	During chemotherapy
After BEP cycle 4	4.4	< 0.1	End of chemotherapy
3‐Month follow‐up	4.6	< 0.1	Surveillance
6‐Month follow‐up	4.3	< 0.1	Surveillance
12‐Month follow‐up	4.2	< 0.1	Surveillance

## 3. Discussion

Testicular embryonal carcinoma, a subtype of NSGCTs, is characterized by its aggressive clinical course and propensity for early metastatic dissemination, most commonly to retroperitoneal lymph nodes. While the majority of reported cases present with elevated tumor markers such as AFP and beta‐*β*‐HCG, a subset of patients, such as the present case, exhibit normal serum markers, complicating the diagnostic process and increasing the risk of misclassification as seminoma or other benign conditions [5, 6]. Literature review reveals similar cases of NSGCT with retroperitoneal lymph node involvement, where clinical suspicion, imaging, and histopathological confirmation were pivotal for diagnosis, particularly in the context of marker‐negative disease [5, 7]. Notably, a case with metastatic embryonal carcinoma to the duodenum also demonstrated normal tumor markers, emphasizing that marker negativity does not exclude aggressive metastatic behavior [5].

Compared with other published cases, our patient’s presentation aligns with the classic features of NSGCT, painless, progressive testicular mass, and retroperitoneal lymph node metastases, yet stands out due to the absence of marker elevation and favorable response to standard therapy (orchiectomy plus BEP chemotherapy) [2, 8]. In contrast, several reports document markedly elevated AFP or *β*‐HCG, often associated with higher tumor burden and, in some instances, atypical paraneoplastic syndromes such as hyperthyroidism [9, 10]. Prognostically, both marker‐positive and marker‐negative NSGCTs can achieve durable remission with multidisciplinary management; however, delayed recognition in marker‐negative cases may increase the risk of advanced disease at diagnosis [5, 11]. Our case underscores the necessity of integrating imaging and immunohistochemical profiling for timely and accurate diagnosis, particularly when tumor markers are unrevealing.

A critical educational point highlighted by this case is the diagnostic challenge posed by testicular embryonal carcinoma when classic tumor markers such as AFP and *β*‐HCG remain within normal ranges. This marker negativity is a well‐recognized diagnostic pitfall, as clinicians may erroneously favor benign or less aggressive etiologies or misclassify the tumor as seminoma, which more frequently presents with normal serum markers [12]. Imaging characteristics, while suggestive, are insufficiently specific to distinguish between various testicular neoplasms; MRI can delineate tumor extent and identify nodular lesions with heterogeneous enhancement, but definitive diagnosis relies on histopathological and immunohistochemical evaluation [13, 14]. Immunohistochemical markers such as CD30, PLAP, and a high Ki‐67 index are critical in differentiating embryonal carcinoma from seminoma and other non‐germ cell tumors [15]. This case thus underscores the necessity of a comprehensive approach, combining clinical, radiologic, and pathologic data, to avoid diagnostic delays that may impact prognosis.

Another teaching point is the pivotal role of a multidisciplinary, protocolized management strategy in optimizing outcomes for advanced testicular embryonal carcinoma, particularly in cases with retroperitoneal lymph node metastasis. The integration of radical orchiectomy followed by adjuvant BEP chemotherapy remains the standard of care and has demonstrated high efficacy and tolerability, even in the context of metastatic disease [16, 17]. While BEP‐related toxicities such as pulmonary fibrosis warrant vigilance, recent evidence suggests that regimen modifications and supportive interventions, including exercise, may mitigate long‐term adverse effects [17]. Importantly, as highlighted in this case, normal tumor markers do not preclude aggressive metastatic behavior or favorable response to standard therapy. The absence of biomarker elevation renders vigilant imaging and pathological confirmation even more essential for early diagnosis and appropriate risk stratification [18]. Ultimately, this case advocates for the early consideration of germ cell malignancy in any young male presenting with a painless testicular mass, regardless of tumor marker profile, and highlights the enduring importance of multidisciplinary collaboration for individualized patient care.

This case serves as a salient reminder of the importance of early recognition and intervention in young males presenting with painless testicular masses, regardless of tumor marker status. The potential for delayed diagnosis in marker‐negative cases highlights the need for heightened clinical vigilance and the routine incorporation of advanced imaging and comprehensive histopathological assessment, including immunohistochemistry, to establish an accurate diagnosis. The present case further demonstrates the critical role of multidisciplinary collaboration, with urologists, radiologists, pathologists, and oncologists jointly contributing to decision‐making and individualized patient management, ultimately leading to favorable outcomes. Furthermore, the successful application of radical orchiectomy and adjuvant BEP chemotherapy in this marker‐negative, metastatic embryonal carcinoma underscores the efficacy of standard treatment protocols, even in atypical clinical scenarios [1]. However, it also reinforces the necessity of close monitoring for treatment‐related toxicity and potential late effects, particularly in young patients who may require long‐term survivorship care planning [17].

A preliminary version of this case report was deposited as a preprint [19] prior to submission. The current version has been revised to include additional clinical details and follows the journal’s policy for previously disseminated content. No part of this manuscript has been published in a peer‐reviewed journal.

## 4. Conclusion

This case highlights the significant diagnostic pitfall posed by normal serum tumor markers in testicular embryonal carcinoma. It emphasizes that for any young male presenting with a testicular mass, clinical assessment, cross‐sectional imaging (including abdominal evaluation), and definitive pathological examination must be integrated. Adherence to this principle within a multidisciplinary team framework is crucial for ensuring timely and accurate diagnosis and effective treatment for such atypical patients.

## Author Contributions

**Panpan Teng:** writing – original draft, investigation, formal analysis, data curation. **Mingxi Yang:** conceptualization. **Shang Song:** conceptualization.

## Funding

This study was supported by The Tongren Municipal Science and Technology Program Project (Tong Municipal Research [2023] No. 25).

## Ethics Statement

Institutional review board (IRB) approval was not required for this case report because it describes a single patient without identifiable information, in accordance with institutional policy and international case report guidelines.

## Consent

Patient data were anonymized and consent was obtained for publication of the data.

## Conflicts of Interest

The authors declare no conflicts of interest.

## Data Availability

The datasets from the current study are available from the corresponding author upon reasonable request.
